# Environmental Influences on Growth and Secondary Metabolite Accumulation in *Eleutherococcus sessiliflorus* Across Korean Cultivation Sites

**DOI:** 10.3390/plants14203175

**Published:** 2025-10-16

**Authors:** Yonghwan Son, Dong Hwan Lee, Jun Hyuk Jang, Hyun-Jun Kim, Ji Ah Kim

**Affiliations:** Forest Medicinal Resources Research Center, National Institute of Forest Science, Yeongju-si 36040, Republic of Korea; thsdydghks@korea.kr (Y.S.); leedh0419@korea.kr (D.H.L.); wnseldu123@korea.kr (J.H.J.); mind4938@korea.kr (H.-J.K.)

**Keywords:** correlation, cultivation, environmental factors, *Eleutherococcus sessiliflorus*, medicinal plants, UPLC-UV

## Abstract

*Eleutherococcus sessiliflorus* is a medicinal shrub widely used in East Asian traditional medicine, yet field-based studies on environmental influences remain limited. In this study, branches from 26 cultivation sites across South Korea were analyzed for relationships among growth traits, soil and climatic conditions, and two major compounds, chlorogenic acid (CGA) and eleutheroside E (EleuE). Growth traits varied widely, with plant height ranging from 1.06 to 4.20 m. CGA content was relatively stable across sites (0.292–0.708 mg/g), while EleuE showed greater variability (0.038–0.264 mg/g). The combined content of CGA and EleuE showed a weak positive correlation with thorn density (*r* = 0.236, *p* = 0.037). Plant height and basal diameter were positively correlated with temperature indices (annual average temperature *r* = 0.410, *p* < 0.001; annual maximum temperature *r* = 0.341, *p* = 0.002), whereas thorn density decreased with soil electrical conductivity, potassium, and magnesium but increased with sand and precipitation. Principal component analysis and correlation networks highlighted distinct clusters separating growth traits from EleuE–environment associations. These findings demonstrate that growth performance in *E. sessiliflorus* is strongly influenced by thermal regimes, while EleuE accumulation responds to soil texture and light availability, providing an empirical foundation for site-specific cultivation strategies and standardized quality management.

## 1. Introduction

*Eleutherococcus sessiliflorus* (Rupr. & Maxim.) S.Y. Hu, a deciduous shrub belonging to the family Araliaceae, is widely distributed across Korea, northeastern China, and Japan. This species usually grows in forested mountain slopes at elevations of 200–1000 m, showing broad ecological adaptability [1,2]. The dried stem or root bark of the genus *Eleutherococcus* is commonly referred to as O-Ga-pi or Wu-jia-pi [3,4].

The stem and root bark of *E. sessiliflorus* have been used for centuries in East Asian medicine to treat a wide range of conditions [5]. According to various traditional sources, it has been prescribed to dispel wind-dampness, strengthen bones and muscles, tonify the liver and kidney, and alleviate fatigue, pain, and other chronic ailments [3,4,6]. These traditional applications highlight its long-standing value as a restorative and health-promoting herbal resource [7], and previous studies have investigated modern pharmacological methods that have revealed that *E. sessiliflorus* exhibits diverse bioactivities, including anti-inflammatory, antioxidant, anticancer, cardioprotective, neuroprotective, and immunomodulatory effects [5,6,7,8,9,10,11,12], as demonstrated in both in vitro and in vivo studies.

These pharmacological effects are largely attributed to the diverse secondary metabolites present in *E. sessiliflorus*, with phytochemical investigations identifying more than 300 compounds, including triterpenoids, phenylpropanoids, flavonoids, and organic acids [5,7,13]. Among the diverse secondary metabolites, eleutheroside E is abundant in the stems and has been highlighted as an important constituent with anti-inflammatory, antidiabetic, and anticancer relevance [7,13,14,15,16]. In contrast, chlorogenic acid is recognized as one of the most abundant phenolic acids in the stem [7,13], and due to its sensitivity to processing conditions and strong antioxidant properties, it has been consistently included in quantitative analyses of genus *Eleutherococcus* [17,18,19]. Together, these constituents highlight the pharmacological and analytical relevance of *E. sessiliflorus* [7,14,17,19,20]. Given the pharmacological and analytical importance of these constituents, recent studies have increasingly emphasized laboratory-oriented approaches, including optimized extraction protocols, advanced analytical methods, and biotechnological strategies such as callus culture and bioreactor production [17,18,21].

In addition to these advances, research within the genus *Eleutherococcus* has actively pursued practice-oriented cultivation evidence to support industrial utilization [5,22,23,24,25]. However, most of this work has concentrated on *E. senticosus*, delineating how specific environmental conditions are linked to the accumulation of target metabolites [26,27,28]. Although one might infer similar patterns for *E. sessiliflorus* as a close congener, comparative data show clear interspecific differences in the contents of eleutherosides B and E and of phenolic acids, and *E. sessiliflorus* exhibits tissue-specific accumulation patterns that make direct transfer of findings from *E. senticosus* inappropriate [19,24]. These considerations lead directly to the next task: a species-focused, field-based assessment of how cultivation environments govern the production of metabolites in *E. sessiliflorus* in order to enable standardized and scalable production [29,30].

Therefore, an assessment specific to *E. sessiliflorus* under field conditions is required to clarify how cultivation environments influence major metabolite accumulation and growth traits, providing a foundation for future standardization. In particular, we test whether differences in environmental factors such as temperature, precipitation, and soil chemistry are associated with consistent shifts in metabolite profiles and growth performance, applying validated analytical methods and multivariate analysis. By establishing species-specific links among environment, major constituents, and growth traits in *E. sessiliflorus*, this study provides empirical evidence that advances ecological understanding and frames priorities for subsequent cultivation research and quality evaluation.

## 2. Results

### 2.1. Growth Characteristics

Growth traits of *E. sessiliflorus* varied significantly among the 26 cultivation sites (Table 1). Plant height ranged from 1.06 ± 0.06 m (Site 21) to 4.20 ± 0.06 m (Site 19), with an overall mean of 2.55 ± 0.10 m. Basal diameter ranged from 1.06 ± 0.07 cm (Site 21) to 8.97 ± 0.87 cm (Site 9), averaging 3.15 ± 0.20 cm. Branch diameter varied between 5.48 ± 0.31 mm (Site 12) and 10.08 ± 0.17 mm (Site 17), with a mean of 7.17 ± 0.16 mm. Fresh branch weight showed the largest variation, from 13.85 ± 0.80 g (Site 20) to 103.94 ± 4.31 g (Site 17), with an average of 42.22 ± 2.10 g. Thorn density was generally low, averaging 1.06 ± 0.12 spines/cm^2^, although Sites 6, 11, and 21 recorded higher values.

### 2.2. Soil and Meteorological Characteristics

Across the 26 cultivation sites, soils were mildly acidic to neutral and predominantly sandy-loam, spanning distinct gradients in fertility and texture (Table 2). Soil pH ranged from 5.27 ± 0.18 at Site 15 to 7.13 ± 0.04 at Site 12. Organic matter content varied between 0.93 ± 0.13% at Site 3 and 7.28 ± 2.20% at Site 25, whereas total nitrogen remained relatively stable at around 0.15 ± 0.01%. Available phosphate ranged widely, from 25.20 ± 2.27 mg/kg at Site 8 to 1531.34 ± 358.98 mg/kg at Site 15. Exchangeable cations also showed broad variation, with calcium contributing most strongly (0.99 ± 0.08–12.10 ± 3.40 cmol^+^/kg), while Mg^2+^, K^+^, and Na^+^ exhibited moderate ranges (Table 2). Cation exchange capacity spanned from 8.99 ± 1.06 at Site 16 to 25.78 ± 2.66 cmol^+^/kg at Site 25. Soil texture was mostly sandy loam, although Sites 9, 13, and 23 had clay fractions exceeding 20%.

Climatic conditions also differed substantially among sites (Table 3). Annual average temperature ranged from 11.6 °C (Site 10) to 16.0 °C (Sites 7, 8), while annual minimum and maximum temperatures reached −20.9 °C (Site 3) and 37.8 °C (Site 5), respectively. Total precipitation varied between 1112.2 mm (Sites 14, 15) and 2585.5 mm (Site 4). Sunshine hours ranged from 1945.3 h (Site 1) to 2466.9 h (Sites 14, 15), and altitude from 23.3 m (Site 9) to 562.4 m (Site 20).

### 2.3. Validation of Analytical Method and Quantification of Major Compounds

The UPLC-UV method developed for the quantification of chlorogenic acid (CGA) and eleutheroside E (EleuE) in *E. sessiliflorus* branches was validated in terms of linearity, sensitivity, precision, and accuracy (Figure S1; Tables S1–S3). Calibration curves for both compounds exhibited excellent linearity (*r*^2^ > 0.9999) across the tested concentration ranges. The limits of detection (LOD) were 0.012 mg/g for CGA and 0.015 mg/g for EleuE, and the limits of quantification (LOQ) were 0.040 mg/g for CGA and 0.050 mg/g for EleuE, demonstrating adequate sensitivity for natural product analysis. Precision, expressed as relative standard deviation (RSD%), was consistently below 2%, indicating good repeatability. Accuracy was further verified through recovery tests, with mean values ranging from 97% to 104%, all within the generally accepted range for analytical validation. These results demonstrate that the applied UPLC-UV method provides reliable and reproducible quantification of CGA and EleuE in *E. sessiliflorus*.

Based on this validated method, quantitative analysis of *E. sessiliflorus* branches was performed to compare compound levels across the 26 cultivation sites (Table 4). CGA was the predominant compound in all populations, with concentrations ranging from 0.292 mg/g (Site 2) to 0.708 mg/g (Site 22) and an overall mean of 0.477 mg/g. Although the difference between the highest and lowest values exceeded twofold, statistical analysis revealed no significant differences in CGA contents among sites. In contrast, EleuE showed greater variability, with concentrations ranging from 0.038 to 0.264 mg/g. Significant differences were observed among sites, leading to the separation of populations into multiple groups. The total content of CGA and EleuE ranged from 0.355 to 0.939 mg/g, confirming differences in combined compound levels among cultivation sites.

### 2.4. Correlation Analysis

**Figure 1:**
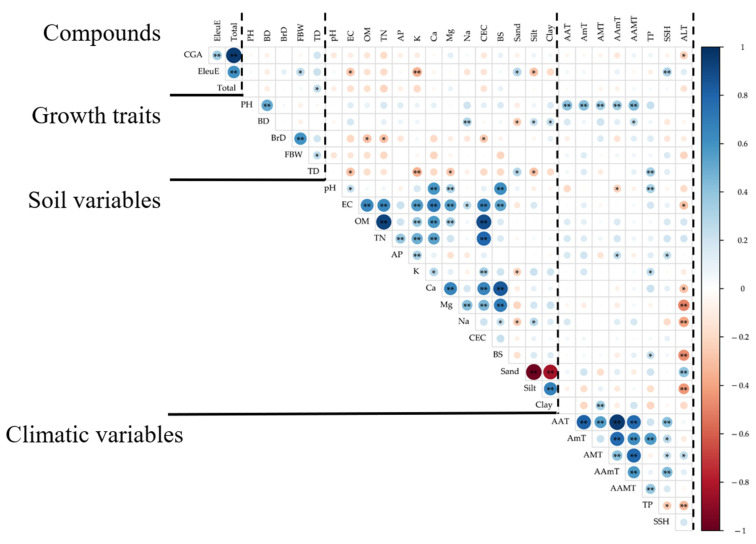
Correlation matrix of compound contents, growth traits, soil, and climate in *Eleutherococcus sessiliflorus* across 26 cultivation sites. Circle size and color represent the strength and direction of Pearson’s correlation (blue = positive, red = negative). Significance levels are indicated by asterisks (* *p* < 0.05, ** *p* < 0.01).

Overall correlation analysis is visualized as in Figure 1, and correlations between growth traits and metabolites were generally weak, with a few notable associations (Table S1). Fresh branch weight correlated positively with EleuE (*r* = 0.258, *p* = 0.023), and the combined content of CGA and EleuE showed a modest positive association with thorn density (*r* = 0.236, *p* = 0.037). CGA showed no significant relationships with growth traits.

Growth traits were more closely tied to environmental variables (Table S2). Plant height increased with AAT (*r* = 0.410, *p* < 0.001), AAMT (*r* = 0.434, *p* < 0.001), AAmT (*r* = 0.352, *p* = 0.002), AMT (*r* = 0.341, *p* = 0.002), and AmT (*r* = 0.417, *p* < 0.001). Basal diameter was positively related to exchangeable Na^+^ (*r* = 0.298, *p* = 0.008). In contrast to overall growth, branch diameter decreased with increasing soil fertility indices, showing negative correlations with organic matter (*r* = −0.284, *p* = 0.012) and total nitrogen (*r* = −0.288, *p* = 0.010). Thorn density displayed the opposite trend to growth, decreasing with higher EC (*r* = −0.281, *p* = 0.013), K^+^ (*r* = −0.345, *p* = 0.002), Mg^2+^ (*r* = −0.264, *p* = 0.020), and silt (*r* = −0.287, *p* = 0.011), but increasing with sand (*r* = 0.277, *p* = 0.014) and total precipitation (*r* = 0.323, *p* = 0.004).

Metabolite–environment relationships were clearest for EleuE that decreased with EC (*r* = −0.266, *p* = 0.019), K^+^ (*r* = −0.346, *p* = 0.002), and silt (*r* = −0.281, *p* = 0.013), but increased with sand (*r* = 0.275, *p* = 0.015) and sunshine hours (SSH; *r* = 0.303, *p* = 0.007) (Table S3). CGA showed weak associations overall, with only a marginal negative correlation with altitude (*r* = −0.229, *p* = 0.044), and the combined content of CGA and EleuE content did not exhibit consistent relationships with climatic variables.

**Figure 2:**
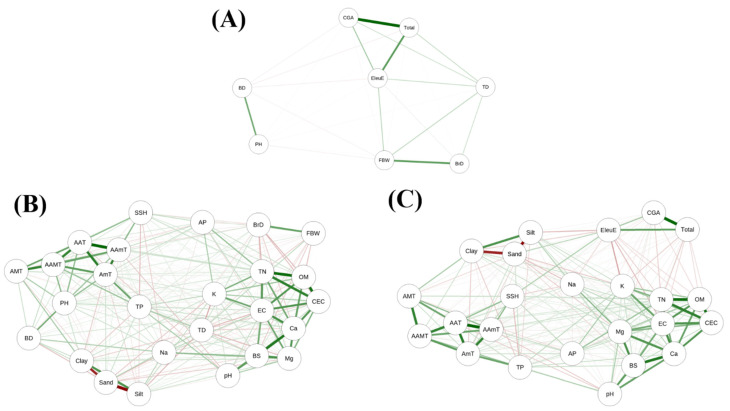
Correlation network models of compound contents, growth traits, and environmental variables in *Eleutherococcus sessiliflorus* across 26 cultivation sites. Panels show (**A**) compound content–growth, (**B**) growth–environment, and (**C**) compound content–environment relationships. Edge color indicates correlation direction (green = positive, red = negative), and edge thickness represents strength.

To visualize the global structure of these associations, a correlation network using significant edges (*p* < 0.05) was constructed (Figure 2). Two distinct clusters emerged. One cluster linked eleutheroside E with soil texture and fertility variables—sand positively and EC, K^+^, and silt negatively—where organic matter and nitrogen also acted as hubs. The other cluster consisted of growth traits, which were strongly intercorrelated and primarily connected to temperature and precipitation. Variables with few connections, such as Na^+^ and total precipitation, were positioned at the periphery. This structure suggests that environmental drivers regulating growth performance and those modulating eleutheroside E accumulation act partly independently in *E. sessiliflorus*.

**Figure 3:**
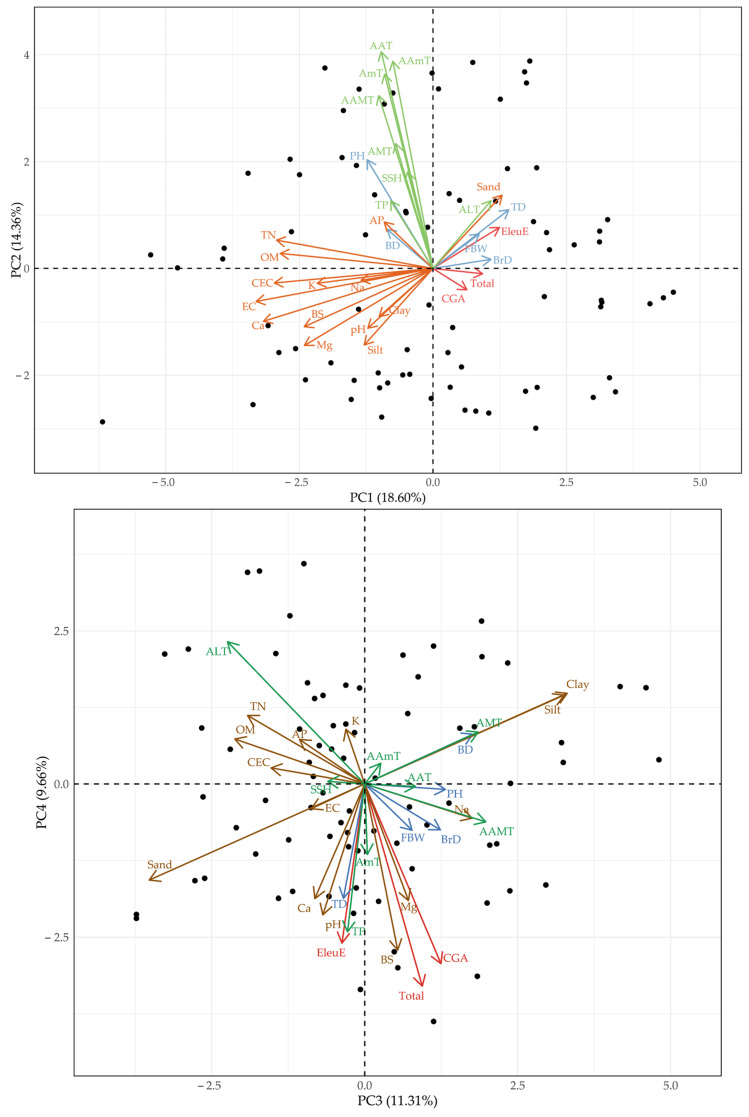
Principal component analysis (PCA) biplots (PC1&PC2, PC3&PC4) of growth traits, soil, climate, and compound contents in *Eleutherococcus sessiliflorus* across 26 cultivation sites. Growth traits are shown in blue, soil in brown, climate in green, and compound contents in red.

Principal component analysis (PCA) provided an integrative view of the association structure (Figure 3). In the PC1&PC2 biplot (37.1%), growth traits (PH, BD, BrD, FBW) clustered with temperature-related variables (AAT, AAmT, AMT, AmT), while EleuE was positioned near TD, Sand, and ALT. In contrast, CGA and Total appeared close to the origin, indicating weak explanatory power in these axes. In the PC3&PC4 biplot (28.5%), EleuE, CGA, and Total were oriented toward soil chemical variables (pH, Ca, Mg, BS), whereas growth traits remained aligned with climatic factors (AAT, AAmT, TP). Notably, TD was located in the same direction as EleuE, suggesting that, unlike PH or BD, this morphological trait shares a response pattern with metabolite accumulation. Together, these results indicate that growth performance and metabolite variation are structured by partially distinct drivers, with TD acting as a bridging trait linked to metabolite responses.

## 3. Discussion

In this study, both growth characteristics and secondary metabolite accumulation in *E. sessiliflorus* varied significantly among cultivation sites. Growth characteristics were more closely associated with temperature-related factors, while EleuE was more sensitive to soil chemistry and texture variables, and also showed a positive correlation with sunshine duration (SSH), indicating light availability may promote its accumulation. By contrast, CGA contents remained relatively stable across sites and showed no consistent associations with environmental variables, supporting its limited sensitivity compared with EleuE. These contrasting responses indicate that growth and secondary metabolite accumulation are regulated by partially distinct, yet interacting sets of environmental drivers, reflecting the complex nature of plant ecological strategies often observed in medicinal species [31].

Across the cultivation sites, overall soil conditions were broadly compatible with tree growth [32]. Within this general pattern, proportions of TN (0.07–0.29%) and Na^+^ were also mostly low (0.02–0.06 cmol^+^/kg). Aside from these consistently low nutrient values, the overall soil properties were in line with habitat descriptions for *E. sessiliflorus* in Korea, which emphasize well-drained sandy-loam soils with slight acidity [33]. At the same time, sites differed markedly in soil fertility and physical properties, particularly in AP, CEC, and BS. These differences indicate distinct nutrient supply regimes among sites [34]. Climatic conditions also varied substantially, especially in temperature-related variables, TP, and elevation. Such climatic variation is well known to affect growth in temperate tree species [35]. Therefore, variation in temperature and soil environments may have contributed to the observed differences, although these relationships should be regarded as correlative rather than causal [36]. Similar variation at the regional level has been reported in other forest medicinal resources, suggesting that differences among sites must be considered in cultivation and management strategies [37].

Within this context, the growth of *E. sessiliflorus* showed statistical associations with climatic and soil environments across cultivation sites. Temperature patterns appeared consistent with the species-specific cardinal temperature range, suggesting that growth occurred under generally favorable thermal conditions [38]. In this study, sandy-loam environments with higher proportions of silt and clay provided soils with adequate water retention while maintaining drainage. These conditions were associated with higher growth characteristics [39,40]. A similar trend was reported in *E. senticosus*, where appropriate water retention was found to enhance growth performance [27]. Among chemical properties, Na^+^ showed a positive correlation with root basal diameter within the non-saline range, indicating that even low Na^+^ availability may have functioned as a relative limiting factor, though this requires further mechanistic confirmation [34]. Interestingly, branch diameter exhibited negative correlations with OM, TN, and CEC. This pattern may reflect a trade-off in resource allocation, consistent with the principle of correlative inhibition [41], whereby height growth is prioritized over branch expansion under competitive conditions [42]. Thorn density showed an opposite pattern to height growth. It decreased with higher soil EC, K^+^, Mg^2+^, and silt contents, but increased with greater sand contents and TP. These findings suggest that prickle density may represent a plastic response to abiotic stressors, particularly under conditions of water instability or drought, although further studies are needed to confirm the underlying mechanisms [43].

With respect to secondary metabolites, eleutheroside E showed negative associations with EC, K^+^, and silt, but a positive association with sand. This indicates that eleutheroside E levels tended to be higher in sites with lower water retention capacity. This agrees with studies reporting that osmotic stress enhances the production of phenylpropanoid and lignan derivatives in medicinal plants, implying that EleuE may serve as a biochemical indicator of site stress [44]. This is consistent with reports that eleutherosides, as phenylpropanoid/lignan derivatives, are strongly influenced by physiological and environmental conditions, and can increase under osmotic stress or jasmonate elicitation [22]. SSH showed a positive correlation with eleutheroside E, indicating that higher light availability may coincide with increased metabolite levels. This relationship should, however, be interpreted as correlative. The result is consistent with Xu et al. (2020) [27], who reported that light environments regulate the accumulation of secondary metabolites in *E. senticosus*. By contrast, chlorogenic acid showed no significant differences among cultivation sites, consistent with previous reports indicating that this compound generally occurs at relatively high levels with narrow variation compared to eleutherosides [17,45,46]. This stability may be related to its role as an early metabolite in the phenylpropanoid pathway, but direct evidence in *E. sessiliflorus* remains limited [47]. Meanwhile, prickle density showed a weak positive correlation with total secondary metabolites, suggesting a possible association between defense morphology and phenylpropanoid accumulation, though this relationship should be interpreted cautiously [48].

In summary, growth of *E. sessiliflorus* was promoted under stable soil conditions with adequate water retention, whereas eleutheroside E and thorn density responded more strongly to variable water and light environments, and chlorogenic acid remained comparatively stable. These results provide evidence of the relationships between environmental factors, growth traits, and secondary metabolites in this species, offering a baseline for quality management. However, as this study was conducted within a single year and limited to cultivation sites in South Korea, inter-annual variation in climatic drivers was not assessed, and the findings should be interpreted within this spatial and temporal framework. Continuous, multi-year studies, together with controlled chamber or greenhouse experiments, will be essential to validate these relationships and provide causal evidence for the patterns observed here. The present work, however, was intended to capture an overall picture of growth traits and metabolite patterns across cultivation sites, thereby providing a foundation for future verification and extension.

## 4. Materials & Methods

### 4.1. Sampling of Plant and Material

**Figure 4:**
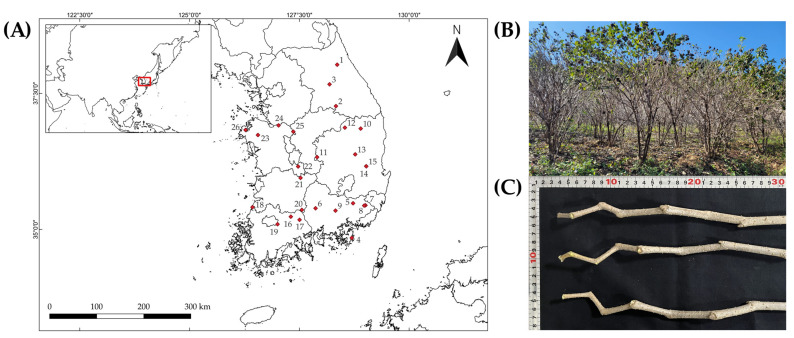
(**A**) Geographic distribution of the 26 cultivation sites of *Eleutherococcus sessiliflorus* in South Korea. (**B**) Representative field view of cultivated plants. (**C**) Sampled branches used for growth and chemical analyses.

Field sampling of *E. sessiliflorus* was carried out in September 2023 across 26 cultivated sites located throughout the Republic of Korea. From each site, three individuals (totaling 78 plants) were randomly selected for sampling (Figure 4; Table S7). Taxonomic identification was conducted by Hyun-Jun Kim of the National Institute of Forest Science, and representative voucher specimens were deposited in the FMRC herbarium (specimen numbers FMRC-B23090001–B23090078). For each plant, a 1 m long branch was excised from the uppermost apical section. Five morphological traits were measured: plant height (PH), basal diameter (BD), branch diameter (BrD), fresh branch weight (FBW), and thorn density (TD). PH and BD were measured with a diameter tape (model SL05001; Shinill Science Co., Paju, Republic of Korea), BrD was measured using a digital vernier caliper (model 500-182-30; Mitutoyo Co., Ltd., Kawasaki, Japan), and FBW was measured using an electronic balance (model HS3200 S; HANSUNG Instrument Co., Gwangmyeong, Republic of Korea). TD was determined by counting the number of thorns present within a standardized 1 cm^2^ surface area.

### 4.2. Sample Preparation

Following the growth trait measurements, branch samples were washed to remove debris and subsequently freeze-dried (PVTFD 20 R; Ilshin Biobase Co., Ltd., Dongducheon, Republic of Korea). The dried material was ground into a fine powder using a grinder (model KSP-35; Korea Medi Co., Ltd., Daegu, Republic of Korea) and passed through an 80-mesh standard sieve prior to extraction. For sample preparation, 500 mg of powdered material was extracted with 10 mL of 100% methanol using an ultrasonic extractor at 30 °C for 1 h (JAC-5020; KODO Co., Ltd., Hwaseong, Republic of Korea). The extracts were centrifuged at 3000 rpm for 10 min (model UM-1258; Labogene, Seoul, Republic of Korea) and filtered through qualitative filter paper (No. 2; Toyo Roshi Kaisha, Ltd., Tokyo, Japan). The filtrates were further purified through a 0.2 μm PTFE syringe filter (Cat. No. 6784-1302; Whatman Co., Maidstone, UK) for UPLC analysis. HPLC-grade water, methanol, and acetonitrile (Avantor Inc., Radnor, PA, USA) were used as solvents for analysis. Analytical standards of eleutheroside E (≥99%, CAS No. 39432-56-9) and chlorogenic acid (≥99%, CAS No. 327-97-9) were purchased from the National Institute for Korean Medicine Development (Sunchang, Republic of Korea).

### 4.3. UPLC Conditions and Validation

To perform the simultaneous analysis of target compounds, UPLC analysis was performed using an ACQUITY UPLC system (Waters Co., Milford, MA, USA) equipped with an ACQUITY UPLC HSS T3 column (2.1 × 100 mm, 1.8 μm, 100 Å; Waters Co., Milford, MA, USA). The column temperature was maintained at 30 °C, and detection was carried out at UV wavelengths of 254 nm for CGA and 220 nm for EleuE using a UV detector. The mobile phase consisted of (A) 0.1% formic acid in water and (B) 0.1% formic acid in acetonitrile. The gradient elution program was as follows: initial, 90% A (10% B); 0–5 min, 90% A (10% B); 5–10 min, 85% A (15% B); 10–20 min, 70% A (30% B); 20–20.1 min, 10% A (90% B); and 20.1–22 min, 10% A (90% B). The flow rate was set to 0.3 mL/min, with an injection volume of 1 μL. All chromatographic operations were controlled, and data were acquired using Empower 3 software (Waters Co., Milford, MA, USA). Each sample was analyzed in triplicate, and results were expressed as mean values.

The analytical method was validated in accordance with the International Conference on Harmonization (ICH) guidelines [49], assessing the parameters of linearity, limit of detection (LOD), limit of quantification (LOQ), precision, and accuracy. Calibration curves for eleutheroside E and chlorogenic acid were established using six concentration levels (5–400 μg/mL). During validation, LOD and LOQ were determined on the solution basis (µg/mL; Table 4) and then converted to matrix-based units (mg/g dry weight, DW) when reporting sample contents. Linearity was excellent for both analytes (*r*^2^ > 0.9999). Precision was evaluated in terms of repeatability as well as intra-day (within a single day) and inter-day (across three consecutive days) variations, expressed as relative standard deviation (RSD, %). Accuracy was determined through recovery assays by comparing the peak areas of spiked and unspiked sample extracts. Each concentration level was analyzed in triplicate, and results are presented as mean values.

### 4.4. Soil Sample and Meteorological Data

At each cultivation site, soil samples were collected in triplicate from the top 20 cm of the soil profile after removing the surface layer. The samples were air-dried under shaded and well-ventilated conditions at room temperature, passed through a 2 mm mesh sieve, and stored for analysis. Soil texture was classified into 12 categories according to the United States Department of Agriculture (USDA) textural classification system [50]. Analyses of physicochemical properties, including pH, electrical conductivity (EC), organic matter (OM), total nitrogen (TN), available phosphate (AP), exchangeable cations (K^+^, Ca^2+^, Mg^2+^, Na^+^), cation exchange capacity (CEC), and base saturation (BS) were conducted following the standard procedures of the Rural Development Administration (RDA, 2023) in the Republic of Korea [51].

Soil pH and EC were determined by mixing 10 g of dried soil with 50 mL of distilled water, followed by measurement with a pH meter and an EC meter, respectively. OM content was analyzed using the Tyurin method, and TN content was determined by the Kjeldahl sulfuric acid digestion method. AP was quantified using the molybdenum blue method with 1-amino-2-naphthol-4-sulfonic acid as the coloring reagent. Exchangeable cations were extracted with 1 N NH_4_ OAc (pH 7.0) and measured by inductively coupled plasma–optical emission spectrometry (ICP–OES), while CEC was calculated from the exchanged ammonium content determined via the Kjeldahl distillation method. Base saturation (BS) was calculated as the percentage of the sum of exchangeable cations relative to CEC. All measurements were performed in triplicate, and values are expressed as means.

Meteorological data corresponding to the year 2023, when the plant materials were collected, were obtained from the Korea Meteorological Administration open portal (data.kma.go.kr) and accessed on 1 January 2025. The variables collected included annual average temperature (AAT), annual average maximum temperature (AAMT), annual average minimum temperature (AAmT), annual maximum temperature (AMT), annual minimum temperature (AmT), total precipitation (TP), and total sunshine hours (SSH).

### 4.5. Statistical Analysis

Statistical analyses were conducted using SPSS software (Version 26; IBM SPSS Statistics, Chicago, IL, USA) and R (Version 4.2.3; RStudio, Posit PBC, Boston, MA, USA). Results are presented as means ± standard error (SE). Differences among groups were tested at *p* < 0.05 using multivariate analysis of variance (MANOVA) followed by Tukey’s test. Pearson’s correlation coefficients were calculated to assess relationships among branch growth traits, compound contents, and environmental variables across cultivation sites. Correlation patterns were visualized as heatmaps using the ggcorrplot2 package and as correlation networks with the qgraph package. Principal component analysis (PCA) was performed with the stats package (prcomp function), and representative biplots were produced using ggplot2.

## 5. Conclusions

This study demonstrates that the growth and metabolite accumulation of *E. sessiliflorus* are strongly influenced by environmental factors. Chlorogenic acid contents were relatively stable across cultivation sites, whereas eleutheroside E showed high variability and greater ecological sensitivity. EleuE was positively linked to fresh branch weight, and the total metabolite content exhibited a weak positive correlation with thorn density. Growth performance was tightly connected to environmental conditions: plant height correlated positively with temperature, while branch diameter and thorn density followed trends opposite to overall growth, being shaped by soil chemistry and water-related factors. These results emphasize that stable production of marker compounds cannot be assumed across diverse environments. Therefore, site-specific ecological variables must be incorporated into cultivation protocols to ensure consistent quality. Collectively, the findings provide a scientific foundation for developing cultivation guidelines and enhancing the sustainable utilization of *E. sessiliflorus* as a medicinal resource.

## Figures and Tables

**Table 1:** Morphological traits of *Eleutherococcus sessiliflorus* branches from 26 cultivation sites in South Korea.

Cultivation Sites	PH	BD	BrD	FBW	TD
	(m)	(cm)	(mm)	(g)	(ea/cm^2^)
1	2.38 ± 0.16 ^defg^	3.37 ± 0.82 ^bcd^	6.40 ± 0.71 ^b^	19.24 ± 1.06 ^de^	0.00 ± 0.00 ^b^
2	2.10 ± 0.04 ^defgh^	1.80 ± 0.06 ^cd^	8.65 ± 0.44 ^ab^	62.24 ± 6.03 ^b^	1.00 ± 0.58 ^ab^
3	2.09 ± 0.21 ^defgh^	3.47 ± 0.13 ^bcd^	8.45 ± 0.59 ^ab^	34.52 ± 6.23 ^bcde^	0.00 ± 0.00 ^b^
4	3.00 ± 0.10 ^abcdef^	2.77 ± 0.03 ^bcd^	7.21 ± 0.98 ^ab^	46.59 ± 9.03 ^bcde^	1.33 ± 0.33 ^ab^
5	3.30 ± 0.70 ^abcd^	2.23 ± 0.48 ^bcd^	7.43 ± 1.08 ^ab^	40.89 ± 13.09 ^bcde^	0.33 ± 0.33 ^ab^
6	3.77 ± 0.20 ^abc^	2.57 ± 0.24 ^bcd^	7.41 ± 0.55 ^ab^	55.66 ± 7.18 ^bc^	2.67 ± 1.20 ^a^
7	3.97 ± 0.15 ^ab^	2.63 ± 0.12 ^bcd^	6.84 ± 0.13 ^ab^	29.12 ± 2.91 ^cde^	1.33 ± 0.67 ^ab^
8	2.13 ± 0.18 ^defgh^	2.93 ± 0.59 ^bcd^	7.10 ± 0.55 ^ab^	30.71 ± 4.91 ^bcde^	0.33 ± 0.33 ^ab^
9	3.80 ± 0.12 ^abc^	8.97 ± 0.87 ^a^	7.05 ± 0.72 ^ab^	49.82 ± 9.96 ^bcd^	1.00 ± 0.58 ^ab^
10	1.83 ± 0.31 ^fgh^	2.33 ± 0.43 ^bcd^	6.56 ± 0.04 ^b^	42.64 ± 3.64 ^bcde^	0.67 ± 0.33 ^ab^
11	2.00 ± 0.15 ^efgh^	2.03 ± 0.09 ^cd^	7.46 ± 0.32 ^ab^	43.97 ± 5.22 ^bcde^	2.67 ± 0.67 ^a^
12	2.27 ± 0.13 ^defgh^	3.40 ± 0.06 ^bcd^	5.48 ± 0.31 ^b^	29.08 ± 3.50 ^cde^	2.00 ± 0.00 ^ab^
13	2.07 ± 0.15 ^defgh^	2.40 ± 0.21 ^bcd^	8.80 ± 1.00 ^ab^	41.71 ± 7.65 ^bcde^	0.67 ± 0.67 ^ab^
14	2.73 ± 0.24 ^bcdef^	4.53 ± 0.98 ^bc^	6.33 ± 0.34 ^b^	49.20 ± 2.18 ^bcd^	1.33 ± 0.33 ^ab^
15	2.60 ± 0.12 ^cdefg^	2.93 ± 0.43 ^bcd^	6.27 ± 0.23 ^b^	51.23 ± 1.74 ^bcd^	0.67 ± 0.33 ^ab^
16	2.37 ± 0.09 ^defg^	5.53 ± 1.91 ^b^	7.36 ± 0.85 ^ab^	23.91 ± 6.77 ^cde^	2.33 ± 0.33 ^ab^
17	1.33 ± 0.09 ^gh^	1.77 ± 0.03 ^cd^	10.08 ± 0.17 ^a^	103.94 ± 4.31 ^a^	1.67 ± 0.67 ^ab^
18	2.65 ± 0.51 ^cdef^	4.57 ± 1.19 ^bc^	6.84 ± 1.04 ^ab^	45.02 ± 6.90 ^bcde^	0.67 ± 0.33 ^ab^
19	4.20 ± 0.06 ^a^	4.93 ± 0.18 ^bc^	8.15 ± 0.84 ^ab^	45.32 ± 1.64 ^bcde^	1.33 ± 0.33 ^ab^
20	2.70 ± 0.21 ^bcdef^	3.07 ± 0.44 ^bcd^	5.72 ± 0.25 ^b^	13.85 ± 0.80 ^e^	0.00 ± 0.00 ^b^
21	1.06 ± 0.06 ^h^	1.06 ± 0.07 ^d^	7.42 ± 0.20 ^ab^	44.71 ± 5.42 ^bcde^	2.67 ± 0.33 ^a^
22	2.60 ± 0.06 ^cdefg^	2.43 ± 0.35 ^bcd^	7.89 ± 0.23 ^ab^	41.09 ± 3.18 ^bcde^	1.67 ± 0.33 ^ab^
23	1.97 ± 0.17 ^fgh^	2.07 ± 0.24 ^cd^	6.25 ± 0.53 ^b^	36.50 ± 4.28 ^bcde^	0.00 ± 0.00 ^b^
24	2.27 ± 0.09 ^defgh^	2.70 ± 0.23 ^bcd^	5.89 ± 0.63 ^b^	33.84 ± 2.31 ^bcde^	0.00 ± 0.00 b
25	1.73 ± 0.03 ^fgh^	1.80 ± 0.06 ^cd^	5.68 ± 0.27 ^b^	35.71 ± 3.01 ^bcde^	0.67 ± 0.67 ^ab^
26	3.27 ± 0.34 ^abcde^	3.73 ± 0.78 ^bcd^	7.71 ± 0.73 ^ab^	47.31 ± 10.26 ^bcd^	0.67 ± 0.33 ^ab^

PH, plant height; BD, basal diameter; BrD, branch diameter; FBW, fresh branch weight; TD, thorn density. Values are expressed as mean ± SE. Different superscript letters within a column indicate significant differences among populations (*p* < 0.05, Tukey’s HSD test).

**Table 2:** Soil physicochemical properties across 26 cultivation sites of *Eleutherococcus sessiliflorus* in South Korea.

No.	pH [1:5]	EC	OM	TN	AP	Exchangeable Cation				CEC	BS	Soil Texture		
						K^+^	Ca^2+^	Mg^2+^	Na^+^			Sand	Silt	Clay
		(dS/m)	(%)		(mg/kg)	(cmol^+^/kg)					(%)	(%)		
1	5.92 ± 0.07 ^defg^	0.24 ± 0.01 ^abc^	3.17 ± 0.36 ^cd^	0.16 ± 0.02 ^bcdefg^	689.90 ± 190.12 ^bcde^	0.45 ± 0.16 ^abc^	4.13 ± 0.80 ^bcdefgh^	0.68 ± 0.15 ^hi^	0.03 ± 0.01 ^c^	15.90 ± 1.34 ^bcdefg^	33.63 ± 5.93 ^defgh^	76.06 ± 1.67 ^abcd^	9.69 ± 0.67 ^fg^	14.25 ± 2.03 ^cd^
2	6.93 ± 0.08 ^ab^	0.32 ± 0.04 ^abc^	2.48 ± 0.17 ^cd^	0.11 ± 0.00 ^defg^	201.14 ± 32.72 ^de^	0.36 ± 0.02 ^abc^	11.82 ± 0.75 ^ab^	2.30 ± 0.37 ^cdef^	0.02 ± 0.01 ^c^	17.38 ± 0.80 ^bcdef^	83.51 ± 0.73 ^ab^	69.72 ± 0.92 ^cdefg^	14.28 ± 0.54 ^bcdefg^	15.99 ± 1.19 ^bcd^
3	6.43 ± 0.04 ^bcde^	0.13 ± 0.01 ^c^	0.93 ± 0.13 ^d^	0.07 ± 0.00 ^g^	38.27 ± 4.51 ^e^	0.13 ± 0.01 ^bc^	2.41 ± 0.34 ^efgh^	0.93 ± 0.05 ^hi^	0.03 ± 0.01 ^c^	11.55 ± 0.52 ^fgh^	30.22 ± 2.25 ^efgh^	64.74 ± 0.45 ^g^	18.76 ± 0.97 ^b^	16.50 ± 1.42 ^abcd^
4	6.43 ± 0.04 ^bcde^	0.35 ± 0.01 ^abc^	5.14 ± 0.28 ^abc^	0.23 ± 0.01 ^ab^	1250.26 ± 36.73 ^ab^	0.69 ± 0.14 ^abc^	10.95 ± 0.14 ^ab^	1.70 ± 0.13 ^cdefgh^	0.09 ± 0.01 ^abc^	20.01 ± 0.61 ^abc^	67.35 ± 3.60 ^abcd^	72.17 ± 0.83 ^abcdefg^	15.28 ± 1.01 ^bcdefg^	12.56 ± 1.71 ^d^
5	6.49 ± 0.06 ^bcde^	0.36 ± 0.02 ^abc^	2.25 ± 0.07 ^cd^	0.11 ± 0.00 ^defg^	135.59 ± 1.61 ^de^	0.47 ± 0.07 ^abc^	9.81 ± 0.54 ^abcd^	3.75 ± 0.38 ^b^	0.04 ± 0.01 ^c^	15.61 ± 0.32 ^bcdefg^	90.25 ± 2.36 ^a^	75.20 ± 0.85 ^abcde^	10.19 ± 0.94 ^efg^	14.62 ± 1.57 ^cd^
6	6.35 ± 0.11 ^cde^	0.21 ± 0.02 ^bc^	3.98 ± 0.64 ^abcd^	0.15 ± 0.02 ^bcdefg^	40.05 ± 26.23 ^e^	0.27 ± 0.05 ^abc^	4.38 ± 0.56 ^cdefgh^	0.67 ± 0.05 ^hi^	0.06 ± 0.02 ^abc^	18.37 ± 0.55 ^bcde^	29.27 ± 2.95 ^efgh^	69.83 ± 0.54 ^bcdefg^	14.16 ± 2.37 ^bcdefg^	16.01 ± 1.84 ^bcd^
7	6.04 ± 0.13 ^defg^	0.29 ± 0.05 ^abc^	2.54 ± 0.58 ^cd^	0.13 ± 0.03 ^bcdefg^	792.25 ± 260.08 ^bcd^	0.27 ± 0.07 ^abc^	8.87 ± 1.17 ^abcdef^	1.26 ± 0.13 ^efghi^	0.04 ± 0.00 ^c^	14.59 ± 1.30 ^cdefgh^	73.59 ± 14.52 ^abc^	65.62 ± 0.76 ^g^	17.06 ± 0.76 ^bcd^	17.32 ± 1.06 ^abcd^
8	6.74 ± 0.02 ^bcde^	0.31 ± 0.03 ^abc^	3.55 ± 0.29 ^bcd^	0.17 ± 0.01 ^bcdefg^	25.20 ± 2.27 ^e^	0.76 ± 0.03 ^ab^	6.28 ± 0.39 ^abcdefgh^	0.97 ± 0.07 ^hi^	0.05 ± 0.01 ^bc^	16.10 ± 0.34 ^bcdefg^	50.25 ± 3.78 ^bcdefg^	76.24 ± 0.43 ^abcd^	10.39 ± 0.35 ^efg^	13.37 ± 0.34 ^cd^
9	6.12 ± 0.05 ^defg^	0.44 ± 0.11 ^ab^	3.54 ± 0.22 ^bcd^	0.19 ± 0.00 ^abcde^	584.05 ± 97.26 ^bcde^	0.56 ± 0.08 ^abc^	9.01 ± 0.61 ^abcd^	2.36 ± 0.18 ^cde^	0.16 ± 0.07 ^ab^	18.74 ± 0.95 ^bcd^	64.44 ± 1.11 ^abcd^	43.69 ± 0.54 ^h^	34.52 ± 1.09 ^a^	21.79 ± 1.52 ^ab^
10	6.43 ± 0.04 ^bcde^	0.33 ± 0.05 ^abc^	2.47 ± 0.25 ^cd^	0.13 ± 0.00 ^bcdefg^	582.29 ± 160.28 ^bcde^	0.56 ± 0.12 ^abc^	7.43 ± 0.36 ^abcdefgh^	1.34 ± 0.08 ^efghi^	0.01 ± 0.00 ^c^	15.54 ± 1.31 ^bcdefg^	60.87 ± 5.25 ^abcde^	71.69 ± 0.87 ^abcdefg^	15.90 ± 0.47 ^bcde^	12.41 ± 0.91 ^d^
11	5.73 ± 0.07 ^fgh^	0.29 ± 0.09 ^abc^	4.23 ± 1.53 ^abcd^	0.18 ± 0.06 ^bcdefg^	378.72 ± 178.37 ^cde^	0.15 ± 0.02 ^bc^	7.88 ± 2.12 ^abcdefg^	1.17 ± 0.05 ^fghi^	0.05 ± 0.01 ^bc^	17.77 ± 3.17 ^bcdef^	50.93 ± 3.48 ^bcdefg^	73.60 ± 3.42 ^abcdef^	13.44 ± 2.26 ^bcdefg^	12.96 ± 1.19 ^d^
12	7.13 ± 0.04 ^a^	0.44 ± 0.02 ^ab^	4.42 ± 0.75 ^abcd^	0.23 ± 0.03 ^ab^	1068.95 ± 326.60 ^abc^	0.62 ± 0.09 ^abc^	11.96 ± 1.12 ^a^	1.63 ± 0.34 ^cdefgh^	0.02 ± 0.00 ^c^	17.72 ± 0.57 ^bcdef^	80.53 ± 8.84 ^abc^	76.02 ± 1.05 ^abcd^	10.71 ± 1.22 ^efg^	13.27 ± 1.65 ^cd^
13	6.18 ± 0.09 ^cdef^	0.30 ± 0.03 ^abc^	3.92 ± 0.40 ^abcd^	0.19 ± 0.02 ^abcde^	579.00 ± 104.48 ^bcde^	0.72 ± 0.36 ^abc^	8.57 ± 0.29 ^abcdefg^	2.26 ± 0.11 ^cdefg^	0.04 ± 0.00 ^c^	16.72 ± 0.28 ^bcdefg^	69.46 ± 2.59 ^abc^	43.39 ± 1.60 ^h^	33.20 ± 0.79 ^a^	23.41 ± 1.51 ^a^
14	5.85 ± 0.07 ^efg^	0.20 ± 0.03 ^bc^	4.57 ± 0.43 ^abcd^	0.20 ± 0.00 ^abcde^	101.96 ± 8.73 ^de^	0.13 ± 0.01 ^bc^	3.88 ± 0.56 ^defgh^	0.76 ± 0.06 ^hi^	0.04 ± 0.01 ^c^	16.63 ± 0.46 ^bcdefg^	28.81 ± 2.98 ^efgh^	69.04 ± 0.72 ^defg^	16.16 ± 1.09 ^bcde^	14.80 ± 0.38 ^bcd^
15	5.27 ± 0.18 ^h^	0.25 ± 0.02 ^abc^	3.20 ± 0.48 ^cd^	0.16 ± 0.02 ^bcdefg^	1531.34 ± 358.98 ^a^	0.43 ± 0.15 ^abc^	1.11 ± 0.25 ^h^	0.25 ± 0.07 ^i^	0.02 ± 0.01 ^c^	14.48 ± 0.23 ^cdefgh^	12.50 ± 2.45 ^h^	75.09 ± 0.99 ^abcde^	10.94 ± 0.35 ^defg^	13.97 ± 0.74 ^cd^
16	6.21 ± 0.12 ^cdef^	0.26 ± 0.02 ^abc^	1.46 ± 0.16 ^d^	0.09 ± 0.01 ^efg^	265.19 ± 85.00 ^de^	0.26 ± 0.05 ^abc^	3.93 ± 0.29 ^defgh^	0.99 ± 0.11 ^hi^	0.01 ± 0.00 ^c^	8.99 ± 1.06 ^h^	59.26 ± 6.80 ^abcde^	77.53 ± 0.56 ^ab^	10.40 ± 1.09 ^efg^	12.07 ± 1.39 ^d^
17	5.58 ± 0.04 ^gh^	0.29 ± 0.02 ^abc^	2.44 ± 0.18 ^cd^	0.11 ± 0.01 ^defg^	287.36 ± 59.18 ^de^	0.48 ± 0.12 ^abc^	2.15 ± 0.11 ^gh^	0.57 ± 0.02 ^hi^	0.03 ± 0.00 ^c^	16.38 ± 0.46 ^bcdefg^	19.66 ± 1.15 ^gh^	76.27 ± 0.39 ^abcd^	11.78 ± 0.14 ^cdefg^	11.95 ± 0.30 ^d^
18	6.06 ± 0.12 ^defg^	0.25 ± 0.03 ^abc^	1.16 ± 0.47 ^d^	0.08 ± 0.02 ^fg^	110.42 ± 78.17 ^de^	0.23 ± 0.03 ^abc^	5.29 ± 1.45 ^bcdefg^	2.74 ± 0.23 ^bc^	0.17 ± 0.01 ^a^	12.06 ± 0.14 ^efgh^	69.67 ± 12.79 ^abc^	68.09 ± 1.27 ^efg^	15.11 ± 0.93 ^bcdef^	16.80 ± 0.77 ^abcd^
19	5.59 ± 0.04 ^gh^	0.30 ± 0.02 ^abc^	3.66 ± 0.10 ^abcd^	0.18 ± 0.00 ^abcdef^	494.81 ± 130.61 ^cde^	0.70 ± 0.21 ^abc^	2.31 ± 0.46 ^fgh^	0.58 ± 0.10 ^hi^	0.02 ± 0.00 ^c^	16.45 ± 0.74 ^bcdefg^	21.72 ± 3.14 ^fgh^	70.83 ± 3.07 ^abcdefg^	13.24 ± 1.70 ^bcdefg^	15.93 ± 1.37 ^bcd^
20	6.03 ± 0.25 ^defg^	0.41 ± 0.07 ^ab^	6.91 ± 1.01 ^ab^	0.29 ± 0.02 ^a^	244.83 ± 106.90 ^de^	0.87 ± 0.17 ^a^	10.51 ± 3.21 ^abc^	1.14 ± 0.20 ^ghi^	0.02 ± 0.00 ^c^	21.79 ± 2.74 ^ab^	55.40 ± 11.62 ^bcdef^	74.40 ± 0.41 ^abcdef^	10.06 ± 0.93 ^efg^	15.55 ± 1.06 ^bcd^
21	5.99 ± 0.09 ^defg^	0.14 ± 0.01 ^c^	1.15 ± 0.03 ^d^	0.08 ± 0.00 ^fg^	299.99 ± 6.37 ^de^	0.25 ± 0.03 ^abc^	0.99 ± 0.08 ^h^	0.20 ± 0.01 ^i^	0.02 ± 0.01 ^c^	10.40 ± 0.15 ^gh^	14.05 ± 0.60 ^h^	76.89 ± 0.69 ^abc^	9.48 ± 0.61 ^fg^	13.63 ± 1.25 ^cd^
22	6.14 ± 0.01 ^defg^	0.15 ± 0.00 ^c^	0.95 ± 0.08 ^d^	0.07 ± 0.00 ^g^	59.70 ± 20.78 ^de^	0.06 ± 0.01 ^c^	6.37 ± 0.40 ^abcdefgh^	1.11 ± 0.13 ^hi^	0.09 ± 0.04 ^abc^	12.29 ± 0.32 ^defgh^	62.07 ± 2.63 ^abcde^	77.86 ± 0.88 ^a^	8.90 ± 0.48 ^g^	13.25 ± 1.28 ^cd^
23	6.11 ± 0.05 ^defg^	0.33 ± 0.08 ^abc^	2.86 ± 0.40 ^cd^	0.14 ± 0.02 ^bcdefg^	636.18 ± 45.70 ^bcde^	0.80 ± 0.26 ^ab^	6.44 ± 1.03 ^abcdefgh^	1.58 ± 0.32 ^defgh^	0.04 ± 0.02 ^c^	15.32 ± 0.16 ^bcdefgh^	57.66 ± 9.47 ^abcde^	46.05 ± 0.27 ^h^	33.71 ± 0.78 ^a^	20.24 ± 1.04 ^abc^
24	5.74 ± 0.10 ^fgh^	0.30 ± 0.02 ^abc^	2.74 ± 0.16 ^cd^	0.12 ± 0.01 ^cdefg^	46.51 ± 6.10 ^e^	0.39 ± 0.02 ^abc^	6.16 ± 1.16 ^abcdefgh^	1.43 ± 0.08 ^defgh^	0.04 ± 0.02 ^c^	16.30 ± 0.55 ^bcdefg^	48.94 ± 6.13 ^cdefg^	48.72 ± 0.87 ^h^	32.85 ± 0.88 ^a^	18.43 ± 0.27 ^abcd^
25	6.01 ± 0.27 ^defgh^	0.49 ± 0.09 ^a^	7.28 ± 2.20 ^a^	0.23 ± 0.03 ^abc^	39.77 ± 6.97 ^e^	0.36 ± 0.09 ^abc^	12.10 ± 3.40 ^a^	5.06 ± 0.58 ^a^	0.13 ± 0.06 ^abc^	25.78 ± 2.66 ^a^	66.94 ± 7.83 ^abcd^	70.52 ± 3.29 ^abcdefg^	14.76 ± 2.31 ^bcdefg^	14.72 ± 1.17 ^cd^
26	6.40 ± 0.08 ^bcde^	0.45 ± 0.07 ^ab^	3.92 ± 0.70 ^abcd^	0.20 ± 0.03 ^abcde^	476.49 ± 44.54 ^cde^	0.68 ± 0.11 ^abc^	8.93 ± 0.45 ^abcde^	2.56 ± 0.22 ^cd^	0.08 ± 0.01 ^abc^	18.70 ± 0.81 ^bcd^	65.58 ± 1.62 ^abcd^	67.12 ± 1.76 ^fg^	17.95 ± 0.34 ^bc^	14.93 ± 2.00 ^bcd^

pH, soil acidity; EC, electrical conductivity; OM, organic matter; TN, total nitrogen; AP, available phosphate; K^+^, potassium; Ca^2+^, calcium; Mg^2+^, magnesium; Na^+^, sodium; CEC, cation exchange capacity; BS, base saturation; Sand, sand content; Silt, silt content; Clay, clay content. Values are expressed as mean ± SE (*n* = 3). Different superscript letters within a column indicate significant differences among populations (*p* < 0.05, Tukey’s HSD test).

**Table 3:** Meteorological variables across 26 cultivation sites of *Eleutherococcus sessiliflorus* in South Korea.

Cultivation Sites (*n* = 3)	AAT	AmT	AMT	AAmT	AAMT	TP	SSH	ALT
	(°C)	(°C)	(°C)	(°C)	(°C)	(mm)	(h)	(m)
1	11.8	−20.8	35.2	6.5	18.1	1225.6	1945.3	419.0
2	12.4	−18.9	36.5	7.0	19.0	1507.5	2264.7	280.0
3	12.1	−20.9	36.8	6.9	18.5	1392.4	1989.4	427.5
4	15.3	−10.4	34.5	11.4	19.6	2585.5	2114.9	43.2
5	15.0	−12.4	37.8	9.6	21.4	1874.0	2327.7	40.9
6	13.9	−13.5	36.4	8.5	20.3	1938.2	2124.3	315.3
7	16.0	−10.6	37.4	11.2	21.7	2172.0	2163.7	264.4
8	16.0	−10.6	37.4	11.2	21.7	2172.0	2163.7	343.6
9	14.5	−15.9	37.6	8.6	21.6	1939.9	2146.7	23.3
10	11.6	−15.5	33.1	6.1	17.9	1828.7	2205.5	254.0
11	13.9	−16.8	36.0	8.7	19.8	1670.6	2331.0	268.1
12	12.4	−17.7	34.8	6.9	18.3	2216.1	2271.7	243.0
13	12.9	−19.2	37.1	6.7	20.3	1575.4	2224.3	209.0
14	15.7	−13.9	36.7	12.0	19.8	1112.2	2466.9	549.0
15	15.7	−13.9	36.7	12.0	19.8	1112.2	2466.9	520.0
16	13.5	−14.2	35.6	8.2	19.8	2139.5	2099.7	202.0
17	13.5	−14.2	35.6	8.2	19.8	2139.5	2099.7	124.9
18	14.1	−16.7	35.3	9.5	19.4	1496.1	2038.8	43.2
19	13.5	−14.2	35.6	8.2	19.8	2139.5	2099.7	391.5
20	13.8	−16.3	37.0	8.6	19.9	1805.7	2143.6	562.4
21	13.0	−17.8	35.2	7.4	19.3	1800.9	2170.2	233.3
22	13.0	−17.8	35.2	7.4	19.3	1800.9	2170.2	269.5
23	13.3	−15.5	35.2	8.6	18.5	1529.7	2220.9	46.9
24	13.0	−18.0	35.0	7.7	19.0	1502.5	2134.1	41.2
25	13.0	−18.0	35.0	7.7	19.0	1502.5	2134.1	201.6
26	13.3	−15.5	35.2	8.6	18.5	1529.7	2220.9	58.8

AAT, annual average temperature; AmT, annual minimum temperature; AMT, annual maximum temperature; AAmT, annual average minimum temperature; AAMT, annual average maximum temperature; TP, total precipitation; SSH, sunshine hours; ALT, altitude. Values correspond to annual statistics for 2023.

**Table 4:** Contents of *Eleutherococcus sessiliflorus* branches across 26 cultivation sites in South Korea.

Cultivation Sites	Chlorogenic Acid	Eleutheroside E	Total
	(mg/g)		
1	0.534 ± 0.041 ^a^	0.129 ± 0.019 ^bcdef^	0.663 ± 0.058 ^ab^
2	0.292 ± 0.022 ^a^	0.157 ± 0.004 ^abcde^	0.449 ± 0.025 ^b^
3	0.345 ± 0.019 ^a^	0.117 ± 0.003 ^cdef^	0.462 ± 0.022 ^b^
4	0.437 ± 0.078 ^a^	0.116 ± 0.008 ^cdef^	0.553 ± 0.071 ^ab^
5	0.629 ± 0.089 ^a^	0.191 ± 0.014 ^abc^	0.820 ± 0.090 ^ab^
6	0.497 ± 0.142 ^a^	0.154 ± 0.019 ^bcde^	0.651 ± 0.160 ^ab^
7	0.549 ± 0.044 ^a^	0.147 ± 0.012 ^bcde^	0.696 ± 0.048 ^ab^
8	0.317 ± 0.099 ^a^	0.062 ± 0.015 ^ef^	0.379 ± 0.114 ^b^
9	0.427 ± 0.093 ^a^	0.058 ± 0.008 ^ef^	0.485 ± 0.100 ^ab^
10	0.431 ± 0.085 ^a^	0.130 ± 0.018 ^bcdef^	0.561 ± 0.101 ^ab^
11	0.504 ± 0.034 ^a^	0.134 ± 0.009 ^bcdef^	0.638 ± 0.025 ^ab^
12	0.449 ± 0.058 ^a^	0.185 ± 0.020 ^abcd^	0.635 ± 0.039 ^ab^
13	0.673 ± 0.068 ^a^	0.131 ± 0.037 ^bcdef^	0.804 ± 0.039 ^ab^
14	0.465 ± 0.008 ^a^	0.264 ± 0.029 ^a^	0.729 ± 0.035 ^ab^
15	0.322 ± 0.017 ^a^	0.131 ± 0.020 ^bcdef^	0.453 ± 0.015 ^b^
16	0.526 ± 0.110 ^a^	0.134 ± 0.012 ^bcdef^	0.660 ± 0.109 ^ab^
17	0.534 ± 0.017 ^a^	0.181 ± 0.017 ^abcd^	0.715 ± 0.033 ^ab^
18	0.551 ± 0.106 ^a^	0.152 ± 0.011 ^bcde^	0.704 ± 0.101 ^ab^
19	0.499 ± 0.025 ^a^	0.136 ± 0.024 ^bcdef^	0.635 ± 0.027 ^ab^
20	0.317 ± 0.036 ^a^	0.038 ± 0.003 ^f^	0.355 ± 0.033 ^b^
21	0.522 ± 0.028 ^a^	0.078 ± 0.015 ^def^	0.600 ± 0.042 ^ab^
22	0.708 ± 0.086 ^a^	0.231 ± 0.047 ^ab^	0.939 ± 0.072 ^a^
23	0.628 ± 0.185 ^a^	0.118 ± 0.002 ^cdef^	0.746 ± 0.183 ^ab^
24	0.427 ± 0.062 ^a^	0.063 ± 0.021 ^ef^	0.491 ± 0.083 ^ab^
25	0.514 ± 0.144 ^a^	0.123 ± 0.027 ^cdef^	0.637 ± 0.171 ^ab^
26	0.303 ± 0.049 ^a^	0.060 ± 0.018 ^ef^	0.363 ± 0.064 ^b^

Values are expressed as mean ± SE. Different superscript letters within a column indicate significant differences among populations at *p* < 0.05 (Tukey’s HSD test).

## Data Availability

The data are not publicly available due to privacy concerns related to the precise locations of cultivation sites; however, the data can be provided by the corresponding author upon reasonable request.

## Supplementary Materials

The following supporting information can be downloaded at: https://www.mdpi.com/article/10.3390/plants14203175/s1, Figure S1: Linearity test (A,C) and UPLC chromatograms (B,D) of eleutheroside E and chlorogenic acid; Table S1: Linear regression, LOD, and LOQ of the two major compounds; Table S2: Precision of the two major compounds for method validation; Table S3: Recoveries of two major compounds; Table S4: Correlation coefficients between major compounds and the growth traits of *Eleutherococcus sessiliflorus*; Table S5: Correlation coefficients between soil and meteorological variables and the growth traits of *E. sessiliflorus*; Table S6: Correlation coefficients between soil and meteorological variables and the concentrations of major compounds in *E. sessiliflorus*; Table S7: Sampling information for cultivation sites of *E. sessiliflorus* in South Korea.
