# Supplementary material for: Environmental Influences on Growth and Secondary Metabolite Accumulation in Eleutherococcus sessiliflorus Across Korean Cultivation Sites
Source: Plants (Basel). 2025 Oct 16;14(20):3175. doi: 10.3390/plants14203175 (PMC12566893; doi:10.3390/plants14203175)
Supplement: Supplementary file 1 [file plants-14-03175-s001.zip › plants-3911592-supplementary.pdf]

Supplementary Data

Environmental Influences on Growth and Secondary Metabolite Accumulation in *Eleutherococcus sessiliflorus* across Korean Cultivation Sites

Yonghwan Son†, Dong Hwan Lee†, Jun Hyuk Jang, Hyun-Jun Kim and Ji Ah Kim *

Forest Medicinal Resources Research Center, National Institute of Forest Science, Yeongju-si 36040, Republic of Korea; thsdydghks@korea.kr (Y.S.); leedh0419@korea.kr (D.H.L.); wnseldu123@korea.kr (J.H.J.)
mind4938@korea.kr (H.-J.K.)

* Correspondence: jiahkim@korea.kr

† These authors contributed equally to this work.

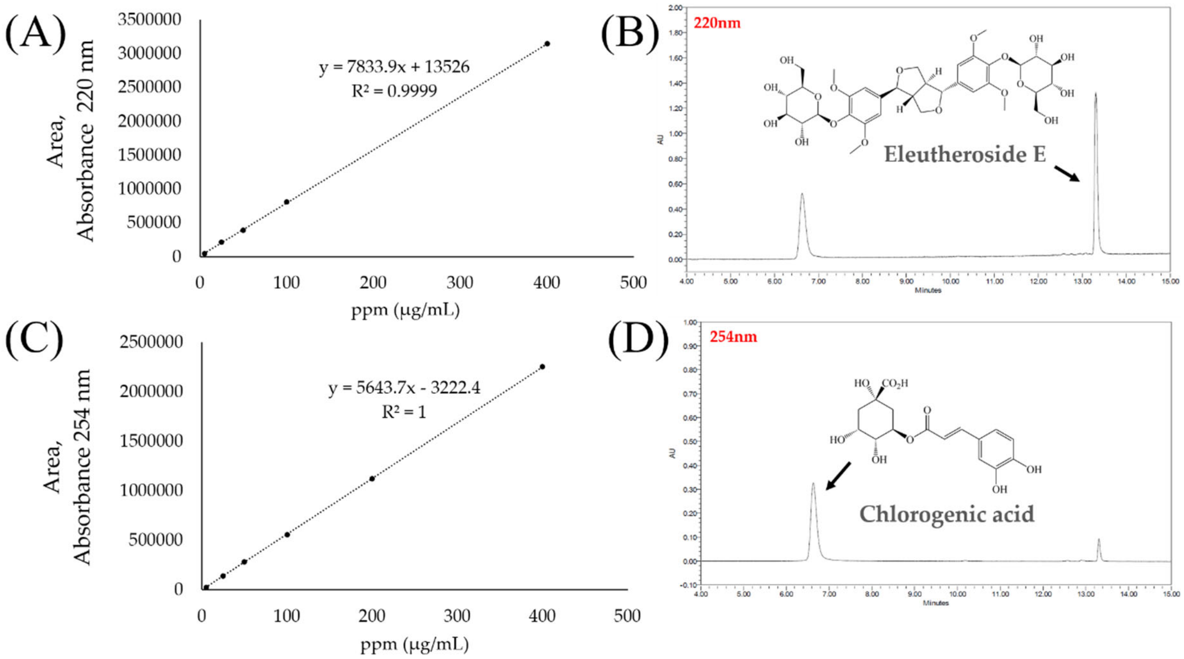

Figure S1. Linearity test (A, C) and UPLC chromatograms (B, D) of eleutheroside E and chlorogenic acid. Calibration curves were constructed at 220 nm for eleutheroside E (A) and 254 nm for chlorogenic acid (C), and representative chromatograms are shown for eleutheroside E (B) and chlorogenic acid (D).

Table S1. Linear regression, LOD, and LOQ of the two major compounds.

Compound	Regression equation	Correlation coefficient (r^2)	Range (μg/mL)	LOD (μg/mL)	LOQ (μg/mL)
Chlorogenic acid	y = 5643.7x – 3222.4	0.9999	5–400	0.04	0.13
Eleutheroside E	y = 7833.9x + 13526	1	5–400	0.02	0.06

Table S2. Precision of the two major compounds for method validation.

Compound	Concentration (μg/mL)	Intra-day [a]		Inter-day [b]	
		Concentration found (μg/mL)	RSD (%)	Concentration found (μg/mL)	RSD (%)
Chlorogenic acid	25	25.01	0.03	25.01	0.03
	50	50.23	0.07	50.23	0.07
	100	99.24	0.08	99.24	0.08
Eleutheroside E	25	25.57	0.41	25.11	0.37
	50	49.09	0.27	49.02	0.72
	100	101.16	0.07	100.18	0.46

[a] Analyzed with three replicates within a single day, $n = 3$; [b] Analyzed on three different days, $n = 3$. RSD, Relative standard deviation.

Table S3. Recoveries of two major compounds.

Compound	Concentration (μg/mL)	Recovery (%)	RSD (%)
Chlorogenic acid	25	101.16	0.29
	50	101.07	0.58
	100	98.01	0.69
Eleutheroside E	25	97.47	0.07
	50	99.79	0.43
	100	98.12	0.22

RSD, Relative standard deviation

Table S4. Correlation coefficients between major compounds and the growth traits of *Eleutherococcus* sessiliflorus

	Correlation coefficient (r)[a]		
**Growth traits**	CGA	EleuE	Total
PH	-0.034<br>(0.768)	0.031<br>(0.790)	-0.019<br>(0.868)
BD	-0.077<br>(0.503)	-0.099<br>(0.389)	-0.096<br>(0.402)
BrD	-0.011<br>(0.926)	0.105<br>(0.361)	0.024<br>(0.837)
FBW	-0.044<br>(0.703)	**0.258***<br>(0.023)	0.043<br>(0.706)
TD	0.198<br>(0.083)	0.219<br>(0.054)	**0.236***<br>(0.037)

PH, plant height; BD, basal diameter; BrD, branch diameter; FBW, fresh branch weight; TD, thorn density; CGA, chlorogenic acid; EleuE, eleutheroside E.

[a] Correlation coefficients (r) are shown, with values in parentheses indicating the corresponding p-values. Significance levels are indicated as follows: * $p < 0.05$; ** $p < 0.01$.

Table S5. Correlation coefficients between soil and meteorological variables and the growth traits of *Eleutherococcus sessiliflorus*

Correlation coefficient (r)[a]											
Soil Properties	PH	BD	BrD	FBW	TD	Meteorological Factors	PH	BD	BrD	FBW	TD
pH	-0.010	-0.050	0.012	-0.139	-0.019	AAT	**0.410****	0.162	-0.089	0.058	0.053
	(0.928)	(0.664)	(0.917)	(0.226)	(0.868)		(0.000)	(0.155)	(0.437)	(0.612)	(0.644)
EC	0.130	0.178	-0.209	-0.132	**-0.281***	AAMT	**0.434****	**0.233***	0.126	0.060	0.101
	0.256	0.119	0.066	0.251	0.013		(0.000)	(0.040)	(0.271)	(0.602)	(0.378)
OM	0.095	-0.005	**-0.284***	-0.164	-0.176	AAmT	**0.352****	0.120	-0.146	0.059	0.003
	0.406	0.963	0.012	0.150	0.123		(0.002)	(0.295)	(0.201)	(0.610)	(0.979)
TN	0.169	0.108	**-0.288***	-0.200	-0.142	AMT	**0.341****	0.208	0.151	-0.025	-0.101
	0.139	0.346	0.010	0.079	0.213		(0.002)	(0.067)	(0.187)	(0.831)	(0.381)
AP	0.131	0.080	-0.137	-0.010	0.006	AmT	**0.417****	0.063	-0.021	0.135	0.144
	0.252	0.486	0.233	0.931	0.961		(0.000)	(0.581)	(0.853)	(0.238)	(0.208)
K	0.081	0.062	0.049	-0.048	**-0.345****	TP	0.222	0.060	0.146	0.060	**0.323****
	0.483	0.591	0.669	0.679	0.002		(0.051)	(0.600)	(0.202)	(0.603)	(0.004)
Ca	0.109	-0.015	-0.199	-0.212	-0.195	SSH	-0.002	-0.122	-0.136	0.150	0.123
	0.341	0.898	0.080	0.062	0.087		(0.984)	(0.286)	(0.235)	(0.190)	(0.284)
Mg	0.030	0.000	-0.095	-0.051	**-0.264***	ALT	0.002	-0.056	-0.108	-0.211	-0.008
	0.797	0.997	0.408	0.656	0.020		(0.986)	(0.626)	(0.345)	(0.063)	(0.941)
Na	0.202	**0.298****	-0.134	0.015	-0.081						
	0.076	0.008	0.242	0.894	0.479						
CEC	0.113	-0.035	**-0.255***	-0.014	-0.197						
	0.326	0.760	0.024	0.904	0.084						
BS	0.151	0.053	-0.065	-0.215	-0.197						
	0.188	0.645	0.573	0.059	0.083						
Sand	-0.098	**-0.243***	0.007	0.000	**0.277***						
	0.394	0.032	0.948	0.997	0.014						
Silt	0.065	**0.223***	-0.056	0.000	**-0.287***						
	0.574	0.049	0.625	0.997	0.011						
Clay	0.153	**0.232***	0.110	0.002	-0.189						
	0.181	0.041	0.340	0.983	0.097						

PH, plant height; BD, basal diameter; BrD, branch diameter; FBW, fresh branch weight; TD, thorn density; EC, electrical conductivity; OM, organic matter; TN, total nitrogen; AP, available phosphate; K, potassium; Ca, calcium; Mg, magnesium; Na, sodium; CEC, cation exchange capacity; BS, base saturation; AAT, annual average temperature; AAMT, annual average maximum temperature; AAmT, annual average minimum temperature; AMT, annual maximum temperature; AmT, annual minimum temperature; TP, total precipitation; SSH, sunshine hours; ALT, altitude.

[a] Correlation coefficients (r) are shown, with values in parentheses indicating the corresponding p-values. Significance levels are indicated as follows: * $p < 0.05$; ** $p < 0.01$.

Table S6. Correlation coefficients between soil and meteorological variables and the concentrations of major compounds in *Eleutherococcus sessiliflorus*

Soil Properties	Correlation coefficient (r)[a] CGA	EleuE	Total	Meteorological Factors	CGA	EleuE	Total
pH	-0.118 (0.302)	0.033 (0.773)	-0.090 (0.433)	AAT	-0.057 (0.618)	0.057 (0.619)	-0.031 (0.789)
EC	-0.099 (0.389)	**-0.266*** (0.019)	-0.167 (0.144)	AAMT	0.071 (0.538)	-0.042 (0.713)	0.047 (0.684)
OM	-0.139 (0.223)	-0.152 (0.183)	-0.166 (0.146)	AAmT	-0.123 (0.281)	0.090 (0.431)	-0.076 (0.506)
TN	-0.184 (0.106)	-0.189 (0.098)	-0.215 (0.058)	AMT	-0.063 (0.585)	-0.025 (0.826)	-0.061 (0.595)
AP	-0.089 (0.438)	-0.030 (0.792)	-0.085 (0.459)	AmT	-0.036 (0.755)	0.051 (0.655)	-0.014 (0.900)
K^+	-0.104 (0.363)	**-0.346**** (0.002)	-0.197 (0.084)	TP	0.068 (0.554)	-0.081 (0.480)	0.032 (0.779)
Ca^{2+}	-0.021 (0.855)	-0.055 (0.635)	-0.035 (0.761)	SSH	-0.067 (0.561)	**0.303**** (0.007)	0.038 (0.741)
Mg^{2+}	0.113 (0.325)	-0.027 (0.812)	0.087 (0.448)	ALT	**-0.229*** (0.044)	0.138 (0.227)	-0.151 (0.187)
Na^+	0.031 (0.788)	-0.142 (0.215)	-0.018 (0.875)				
CEC	-0.125 (0.276)	-0.141 (0.217)	-0.150 (0.190)				
BS	0.091 (0.427)	0.041 (0.718)	0.090 (0.432)				
Sand	-0.074 (0.518)	**0.275*** (0.015)	0.023 (0.842)				
Silt	0.073 (0.525)	**-0.281*** (0.013)	-0.026 (0.821)				
Clay	0.060 (0.604)	-0.194 (0.089)	-0.010 (0.930)				

CGA, chlorogenic acid; EleuE, eleutheroside E; EC, electrical conductivity; OM, organic matter; TN, total nitrogen; AP, available phosphate; K, potassium; Ca, calcium; Mg, magnesium; Na, sodium; CEC, cation exchange capacity; BS, base saturation; AAT, annual average temperature; AAMT, annual average maximum temperature; AAmT, annual average minimum temperature; AMT, annual maximum temperature; AmT, annual minimum temperature; TP, total precipitation; SSH, sunshine hours; ALT, altitude.

[a] Correlation coefficients (r) are shown, with values in parentheses indicating the corresponding p-values. Significance levels are indicated as follows: * $p < 0.05$; ** $p < 0.01$.

Table S7. Sampling information for cultivation sites of *Eleutherococcus sessiliflorus* in South Korea

No.	Locality	Latitude	Longitude	Altitude(m)
1	Inje-gun	38°00'	128°21'	419.0
2	Yeongwol-gun	37°16'	128°20'	280.0
3	Hongcheon-gun	37°39'	128°11'	427.5
4	Geoje-si	34°50'	128°41'	43.2
5	Miryang-si	35°29'	128°43'	40.9
6	Sancheong-gun	35°23'	127°52'	315.3
7	Yangsan-si 1	35°26'	129°00'	264.4
8	Yangsan-si 2	35°26'	128°58'	343.6
9	Haman-gun	35°21'	128°19'	23.3
10	Bonghwa-gun	36°51'	128°53'	254.0
11	Sangju-si	36°20'	127°54'	268.1
12	Yeongju-si	36°52'	128°32'	243.0
13	Uiseong-gun	36°23'	128°46'	209.0
14	Pohang-si 1	36°10'	129°01'	549.0
15	Pohang-si 2	36°10'	129°01'	520.0
16	Gokseong-gun	35°14'	127°18'	202.0
17	Gurye-si	35°10'	127°30'	124.9
18	Yeonggwang-gun	35°24'	126°26'	43.2
19	Hwasun-gun	35°05'	127°00'	391.5
20	Namwon-si	35°21'	127°33'	562.4
21	Jinan-gun	35°57'	127°31'	233.3
22	Geumsan-gun	36°09'	127°28'	269.5
23	Seosan-si	36°44'	126°33'	46.9
24	Asan-si	36°55'	127°01'	41.2
25	Cheonan-si	36°48'	127°21'	201.6
26	Taean-gun	36°50'	126°16'	58.8
